# Developmental history and stress responsiveness are related to response inhibition, but not judgement bias, in a cohort of European starlings (*Sturnus vulgaris*)

**DOI:** 10.1007/s10071-018-1226-7

**Published:** 2018-11-23

**Authors:** Annie Gott, Clare Andrews, Tom Bedford, Daniel Nettle, Melissa Bateson

**Affiliations:** Centre for Behaviour and Evolution and Institute of Neuroscience, Newcastle University, Newcastle, UK

**Keywords:** Judgement bias, Early-life adversity, Affect, Starlings, *Sturnus vulgaris*, Avian cognition

## Abstract

Judgement bias tasks are designed to provide markers of affective states. A recent study of European starlings (*Sturnus vulgaris*) demonstrated modest familial effects on judgement bias performance, and found that adverse early experience and developmental telomere attrition (an integrative marker of biological age) both affected judgement bias. Other research has shown that corticosterone levels affect judgement bias. Here, we investigated judgement bias using a modified Go/No Go task in a new cohort of starlings (*n* = 31) hand-reared under different early-life conditions. We also measured baseline corticosterone and the corticosterone response to acute stress in the same individuals. We found evidence for familial effects on judgement bias, of a similar magnitude to the previous study. We found no evidence that developmental treatments or developmental telomere attrition were related to judgement bias per se. We did, however, find that birds that experienced the most benign developmental conditions, and birds with the greatest developmental telomere attrition, were significantly faster to probe the learned unrewarded stimulus. We also found that the birds whose corticosterone levels were faster to return towards baseline after an acute stressor were slower to probe the learned unrewarded stimulus. Our results illustrate the potential complexities of relationships between early-life experience, stress and affectively mediated decision making. For judgement bias tasks, they demonstrate the importance of clearly distinguishing factors that affect patterns of responding to the learned stimuli (i.e. response inhibition in the case of the Go/No Go design) from factors that influence judgements under ambiguity.

**Electronic supplementary material:**

The online version of this article (10.1007/s10071-018-1226-7) contains supplementary material, which is available to authorized users.

## Introduction

In humans, exposure to early-life adversity has been repeatedly linked to the development of affective disorders such as depression and anxiety (Parker et al. 1995; Sadowski et al. 1999; Kendler et al. 2002; Heim et al. 2008). Recent attempts to model the association between early-life adversity and adult negative affect in non-human animals have used ‘judgement bias’ paradigms (Harding et al. 2004; Mendl et al. 2009; Hales et al. 2014; Roelofs et al. 2016). These are also known as ‘cognitive bias’ paradigms, but the term ‘judgement bias’ is more precise, since the cognitive bias investigated is specifically in judgement rather than, for example, attention or memory. Judgement bias paradigms are motivated by the fact that depressed and anxious humans tend to interpret ambiguous information in the more negative of its possible ways (Ouimet et al. 2009; Everaert et al. 2017). These biases are not restricted to clinically diagnosed disorders, but are also found in individuals with sub-clinical symptoms and those in remission (Everaert et al. 2017), suggesting that judgement biases index chronic negative affect. There is some evidence that adverse childhood experiences are specifically linked to the negative cognitive biases found in depression (Günther et al. 2015).

In animal judgement bias experiments, subjects are trained to associate one stimulus (the positive stimulus) with a high-value reward and a second stimulus (the negative stimulus) with a punisher, lower value reward, or absence of reward. They are then tested with stimuli intermediate between the positive and negative learned stimuli on some continuous dimension. Treating the intermediate stimuli similar to the positive learned stimulus is taken to indicate ‘optimism’, whereas treating them similar to the negative learned stimuli is taken to indicate ‘pessimism’, and hence analogous to cognition during depressed or anxious mood in humans. Judgement bias paradigms have been widely used across many species (see Mendl et al. 2009; Baciadonna and McElligott 2015; Roelofs et al. 2016 for reviews). An emerging issue is that effects can sometimes be in the opposite direction to the general hypothesis, suggesting that the task may not be measuring ‘pessimism’, but some other motivational change. For studies of judgement bias in relation to early-life adversity, the general hypothesis is that adverse early conditions will produce increased ‘pessimism’, and this is the pattern observed, for example, in a study of juvenile unpredictable stress exposure in rats (Chaby et al. 2013). However, Brydges et al. (2012) obtained the opposite result in rats (greater ‘optimism’ in individuals exposed to juvenile stress). Since the rewards in animal judgement bias experiments involve food, Brydges et al. suggested that the task may have been measuring heightened food motivation, rather than cognitive optimism.

To date, there is only one study exploring the effect of early-life adversity on judgement bias in birds. Bateson et al. (2015) applied a judgement bias design originally developed for the starling by Bateson and Matheson (2007). They trained 3-month-old European starlings (*Sturnus vulgaris*) to associate a grey-shaded lid with the presence of a palatable mealworm, and a lid of a different shade of grey with the presence of a toxic quinine-injected mealworm. They were then tested with stimuli of an intermediate shade of grey. Early in their development, the birds had been experimentally assigned, via cross-fostering, to different levels of intra-brood competition in the nest. Individuals raised in large broods were faster to probe the learned stimuli, but once this was controlled for, they showed relatively longer latencies to remove ambiguous lids. This suggests that early-life competition causes negative judgement bias, as well as altered decision-making towards an unambiguous food source (i.e. the learned lids). Bateson et al. (2015) also measured developmental telomere attrition, a marker of biological age. Birds that had experienced greater developmental telomere attrition showed relatively shorter latencies to remove ambiguous lids. This association was significant after controlling for brood competition, and was in the opposite direction. That is, greater intra-brood competition apparently produced greater ‘pessimism’, supporting the general hypothesis, whilst greater developmental telomere attrition produced greater ‘optimism’, more in line with the rat results of Brydges et al. (2012). Bateson et al. (2015) tentatively concluded that social experience (intra-brood competition) and somatic state (developmental telomere attrition), though both consequences of development, may influence decision-making in different ways: adverse social experience sets up negative expectations about rewards, whilst poor somatic state leads individuals to be bolder or ‘desperate’ for food.

The results of Bateson et al. (2015) require replication. Moreover, there were some limitations to both the judgement bias task, and the developmental manipulation, in that study. The judgement bias task was designed to contrast a high-value reward (palatable mealworm) with a punisher (quinine-injected mealworm). However, most birds in the experiment continued to consume the quinine-injected mealworms, albeit with slower latencies than the palatable mealworms. Thus, the findings might reflect treatment-related differences in the acceptability of quinine mealworms as food as much as differences in general judgement bias. Indeed, we have previously found that early experience can influence dietary selectivity in starlings (Bloxham et al. 2014). Thus, here we amended the task to feature a learned contrast between a high-value reward (palatable mealworm) and no reward at all. Such designs have been widely used for judgement bias tasks in other species (e.g. Bateson and Nettle 2015). We chose no reward for the negative option, rather than a lower value reward, as the contrast between reward and no reward is both faster to train and produces larger behavioural differences than the contrast between a larger and a smaller reward.

In terms of the developmental manipulation, Bateson et al. (2015) cross-fostered nestlings to broods of either two (low-competition treatment) or seven (high-competition treatment), from day 4 to day 15 of life, whereafter the birds were reared in the laboratory. However, the results of the manipulation were variable: in some high-competition broods, some competitors died, reducing the experienced level of competition; and in some large broods, the focal individuals were (by chance) the largest in the brood and hence buffered from the negative effects of competition. Moreover, altering the size of the brood confounds two potentially separable sources of adversity: lower per capita food supply, and greater begging effort to obtain food from parents. The birds we used in the current study derived from a better-controlled, hand-rearing based manipulation of early-life adversity (see Nettle et al. 2017 for full details). This paradigm used a two-by-two factorial design in which we independently manipulated the Amount of food received (either Plenty, effectively ad libitum, or Lean, around 73% of the Plenty ration to mimic the slowest growth rates observed in wild nests); and begging Effort (either Easy, fed in response to begging at every nest visit, or Hard, being made to beg for the same length of time again per day by stimulation without feeding on ‘sham’ feeding visits). By hand-rearing the birds, we were able to ensure that every individual received the exact schedule of experience envisaged in the design.

The first main objective of the present study was to conceptually replicate the findings of Bateson et al. (2015), of opposite effects of early adverse experience and developmental telomere attrition on judgement bias in adulthood. Based on the previous results, we predicted that more adverse early experience (Lean Amount or Hard Effort, or both) would produce lower latencies to probe the learned negative stimulus, but relatively higher latencies to probe the ambiguous intermediate stimuli (i.e. greater ‘pessimism’). Simultaneously, we predicted that greater developmental telomere attrition would be associated with relatively lower latencies to probe the ambiguous stimuli (i.e. greater ‘optimism’). In addition, Bateson et al. (2015) observed that members of the same genetic family were more similar to one another than chance in terms of response to ambiguous stimuli, but not in terms of speed to probe the learned stimuli. Since our design also included cohorts of siblings, we were also able to test this. Our design was in fact stronger than theirs for testing for prenatal and genetic effects, since every bird was raised in a different artificial nest from all of its siblings.

The second major objective of this study was to relate performance in the judgement bias experiment to functioning of the hypothalamic–pituitary–adrenal (HPA) stress response system. The association between mood disorders and early-life adversity in humans is thought to be mediated by persistent changes in the HPA axis and circulating glucocorticoid hormone concentrations (Holsboer 2000; Heim et al. 2008). Two recent experimental studies, one in rats and one in chickens, have suggested that increasing levels of the glucocorticoid hormone corticosterone (CORT) may cause a switch to more ‘pessimistic’ performance on judgement bias tasks (Enkel et al. 2010; Iyasere et al. 2017). In the current cohort of birds, we did not manipulate CORT levels experimentally. However, we had measured individuals’ baseline CORT and CORT responses to an acute capture-handling-restraint stressor approximately 3 months prior to the present experiment (data previously published in Gott et al. 2018). This allowed us to ask whether individual differences in judgement bias were related to individual differences in baseline CORT or the magnitude of the CORT response. We predicted that higher baseline CORT, and/or a larger CORT response would be associated with greater ‘pessimism’ in the judgement bias experiment.

## Materials and methods

### Study animals and husbandry

Subjects in this study were 31 adult European starlings (*S. vulgaris*; 16 male) from a cohort of 32 hatched in May 2014 that were subject to a developmental manipulation described previously (Nettle et al. 2017). Briefly, four experimental groups (Plenty-Easy, Plenty-Hard, Lean-Easy or Lean-Hard) were hand-reared in artificial nests (two nests of four nestlings per experimental group), receiving 9 feeds a day. Eight wild nests each provided one member of each experimental group. Sex is not phenotypically obvious in starling nestlings, and subsequent genetic sexing revealed that sex was not well balanced across groups (numbers of males: Plenty-Easy 4; Plenty-Hard 7; Lean-Easy 0; Lean-Hard 8).

The manipulation was applied from day 5 to day 15 post-hatch. In the Plenty groups, birds were fed *ad libitum* in 9 feeds per day, whilst the Lean groups were fed approximately 73% of the corresponding Plenty group’s intake. Hard groups were ‘visited’ (by the researchers) an additional nine times to Easy groups. During these visits, nestlings were stimulated to beg for approximately the same duration as on a real feeding visit (2 min), but no food was delivered. Thus, birds in the Hard conditions begged for around twice as long per day as those in the Easy conditions, and received food on 50%, rather than 100%, of ‘parental’ visits.

After the manipulation, birds were fed ad libitum until fledging (around day 21). After fledging, they were housed together in mixed-treatment indoor aviaries in groups of up to 20 (215 × 340 × 220 cm; 18 °C, 40% humidity), with *ad libitum* food and water. Birds were maintained in non-breeding condition by a constant 15 h light: 9 h dark cycle. The present judgement bias experiment was conducted between October 2014 and February 2015 (mean age at beginning of experiment 217 days). Four replicates of eight birds (two complete genetic families per replicate) were caught from indoor aviary housing and moved to the experimental room (18 °C, 35% humidity, 14:10 light cycle). Here, birds were individually caged (75 × 45 × 45 cm) with access to two perches and two water drinkers for the entirety of the experiment. *Ad libitum* food and water baths were present at all times except during experimental sessions, which ran for approximately 2 h every morning. Food was removed from cages 1 h before sessions began. Birds remained in cages for between 2 and 3 weeks depending on the time taken for each replicate to learn the task. Birds were able to see and hear others at all times except during the experimental sessions, when they were visually isolated using curtains. When a replicate had completed the experiment, the birds were returned to the aviary and replaced with the next two families. Birds were weighed upon entry to and exit from the experiment.

### Developmental telomere attrition

Nestlings were blood-sampled on days 5 and 56 by puncture of an alar or metatarsal vein and collection of approximately 70 µl of blood using a heparinised microcapillary tube. Telomere length was measured in erythrocyte DNA using a real-time PCR amplification method adapted for use in birds (Cawthon 2002; Criscuolo et al. 2009). As described previously (Nettle et al. 2017), we used the two telomere length estimates to gain a single-number measure of telomere shortening, ΔTL. ΔTL was standardised using a formula that corrects for regression to the mean (Verhulst et al. 2013). All birds’ telomeres shortened over the period from day 5 to day 56; a ΔTL of zero represents the average amount of change in the sample, and a negative number indicates more extreme shortening. Due to failed assays (see Nettle et al. 2017), the realised sample size for ΔTL was 27, and hence this is the sample size for analyses involving ΔTL.

### Corticosterone measurement

CORT profiles were measured from the same 31 birds when they were aged 127–134 days (methods described in Gott et al. 2018; the data used here are ‘age point 1’ from that study as this is the closest in age to the judgement bias experiment). Briefly, we used a standardised capture-handling-restraint protocol employed previously in European starlings (Andrews et al. 2017). Birds had not been disturbed for 2 h prior to sampling. Birds were rapidly caught from their cages and transferred immediately to an adjacent room. Approximately 120 µl of a baseline blood sample was collected within 3 min (mean time to baseline sample ± SD, 92.1 ± 18.1 s). Birds were placed in drawstring cloth bags and further samples were taken at 15 and 30 min after the initial disturbance. CORT levels in plasma extracts were quantified using radioimmunoassay (RIA) previously validated in European starlings (Buchanan et al. 2003). The dynamics of the stress response were characterised by three variables: (a) baseline CORT concentration (first sample); (b) peak CORT concentration (higher of second and third samples); (c) ΔCORT, the change in CORT between 15 and 30 min. A positive value of ΔCORT indicates an increase in CORT concentration between 15 and 30 min, and a negative value a decrease. Realised sample size for CORT measurements was 30, and hence this is the sample size for analyses involving CORT variables.

### Judgement bias task

Judgement bias was assessed using an adapted Go/No-Go task. Birds were an average of 217 days old at the start of the experiment. The methods used were similar to those presented in Bateson et al. (2015), except that the outcome associated with the NEG stimulus was an empty dish rather than a quinine-injected mealworm. Birds were initially trained (‘lid-probing training’ phase) to remove a lid covering a Petri dish glued to a plastic white tile containing an obscured mealworm within a 60-s time period. Birds were given a maximum of 16 trials per day. One bird (Plenty-Hard treatment) was excluded after 30 unsuccessful training trials, leaving 31 birds to complete the experiment. This is the same as the number of birds in the judgement bias experiment by Bateson et al. (2015).

Once birds could successfully remove lids, discriminative stimuli in the form of different coloured lids were introduced, indicating the presence (POS) or absence (NEG) of the worm (‘discrimination training’ phase). The colours of lids were achromatic percentages of grey scale (20% or 60% printed in black on white laminated card). Each replicate was assigned either 20% or 60% grey as the POS and NEG stimuli, respectively, counterbalanced across replicates. Birds were given 16 trials per day, in which a dish with either a POS or a NEG lid (8 trials of each in pseudorandom order) was presented, followed by a 4-min inter-trial interval. Birds were required to demonstrate successful learning of the difference between POS and NEG lids in the form of significantly faster latencies to remove the POS lids when compared to the NEG lids (assessed using non-parametric Mann–Whitney *U* tests on each day’s data for each bird). To progress to the test stage, birds had to show successful discrimination by responding significantly faster to POS lids than NEG lids on two consecutive days of trials. There then followed a partial reinforcement training phase, where the worm was only present on 75% of POS trials. The aim of this phase was to train the birds that not all trials were reinforced, to slow down extinction of lid probing in the subsequent test phase, where intermediates were presented in extinction. The partial reinforcement phase had a minimum duration of 2 days, and to progress to the next phase, significant discrimination between POS and NEG again had to be demonstrated.

To assess judgement bias, birds underwent 4 days of ‘test phase’ sessions featuring both the trained stimuli (POS and NEG lids) and ambiguous stimuli intermediate between them (NEARPOS, MID, NEARNEG lids − 30%, 40% and 50% grey scale tested in extinction). Each day’s session consisted of six reinforced POS trials and twelve unreinforced trials: six NEG, two NEARPOS, two MID, and two NEARNEG, again with an inter-trial interval of 4 min. The order of trials was pseudorandom, subject to the constraint that the very first trial was always POS. Successful discrimination was checked at the end of the testing stage by comparing the latencies to probe the POS and NEG lids combined over the four testing days using Mann–Whitney *U* tests for each bird. All 31 birds maintained successful discrimination throughout judgement bias testing and all data were subsequently used in analyses.

### Data collection and statistical analysis

Behaviour during trials was recorded on two video cameras, each capturing four cages. Birds were identifiable by individual colour rings, but the experimenter remained blind to the treatment group to which they belonged. Latencies to touch the lids with the beak and to subsequently eat the mealworms were manually scored by an experimenter after each day of the experiment from video recordings. Trials were deemed to have started when the experimenter’s hand had fully left the cage. If the bird did not touch the lid or the worm within the 60 s time period, then the latencies were recorded as 61.

Latencies to probe lids were bounded between 0 and 61 s. Censoring adjustments were not made as inspection of model residuals showed satisfactory distributions, and most trials (62%) produced a latency less than 61. There were two relatively extreme values of ΔTL: we examined whether these had unusually high leverage for significant results involving that variable (see “Results”).

Data were analysed in R (R Core Development Team 2018). For the analyses of developmental treatments and ΔTL, we used linear mixed effects models implemented with packages ‘afex’ (Singmann et al. 2018) and ‘lme4’ (Bates et al. 2015), with the individual trial as the unit of analysis. We closely followed the analysis strategy of Bateson et al. (2015). This strategy fits separate models for the birds’ latencies on the learned stimuli, and their latencies on the ambiguous stimuli after controlling for their mean latency on the learned stimuli. The former provides a measure of overall motivation; it is the latter that is the direct test of judgement bias. Latency to probe was log-transformed for these analyses. Models contained random terms for bird (because of repeated measures) and natal family (because the cohort consisted of quartets of siblings). Developmental treatments and ΔTL were included simultaneously, as in Bateson et al. (2015). Sex was not included as an additional covariate, to remain close to the analysis of the earlier paper, and because sex did not significantly predict latency to probe (see Supplementary Analyses). Body condition was significantly related to latency to probe (see Supplementary Analyses); however, alterations in body condition are a long-term consequence of the developmental manipulation in this cohort (Dunn et al. 2018), and thus it is inappropriate to control for body condition when assessing the causal impact of developmental treatment on latency. Instead, we consider body condition as a possible mediator of treatment effects (see “Results”).

Parameter estimation was by maximum likelihood, except for estimating familial effects which used restricted maximum likelihood. For fixed effects, type-III significance tests were obtained using Satterthwaite’s method. This method provides *t* tests for each fixed effect, which may have fractional degrees of freedom due to the clustering of the data. Significance tests for the random effect of family were obtained by comparing the deviance of a model with the random effect included versus omitted.

When relating the CORT variables to judgement bias performance, we cannot assign the variables to outcome and predictor roles: both sets of variables are roughly contemporaneous measures of individual differences. Thus, we used correlations for these analyses, with the bird as the unit of analysis. We characterised judgement bias performance per bird using the mean latency to probe the positive learned stimulus (henceforth positive latency), the mean latency to probe the negative stimulus (negative latency), and a pessimism index (again following Bateson et al. 2015). This index was calculated as: (mean latency to probe ambiguous stimuli − positive latency)/(negative latency − positive latency). Thus, a pessimism index of zero indicates that the bird treated the ambiguous stimuli exactly like the positive stimulus; and a pessimism index of one indicates that the bird treated the ambiguous stimuli exactly like the negative stimulus. The three judgement bias variables were correlated with each of baseline CORT, peak CORT and ΔCORT. Correlations involving peak CORT were partial, controlling for baseline CORT, and correlations involving ΔCORT were partial, controlling for CORT at 15 min (see Gott et al. 2018). An alpha level of significance of *P* < 0.05 was used throughout.

### Data availability

The datasets generated during the current study, and the R code used to analyse them, are publicly available via the Zenodo repository at: 10.5281/zenodo.1412327.

## Results

### Training and discrimination learning

Birds took an average of 10.8 ± 4.9 (mean ± SD, *n* = 31) trials to complete lid-probing training. Number of trials to meet criterion was not significantly associated with developmental treatments or ΔTL, or any CORT variable (see Supplementary Analyses). All 31 birds learnt the association between the POS stimulus and the presence of a mealworm, and between the NEG stimulus and the empty dish. Birds took an average of 3.84 ± 1.53 days (mean ± SD) to successfully complete the discrimination training phase, defined as probing POS lids significantly slower than NEG lids over two consecutive days (Mann–Whitney tests *P* < 0.05). We found no significant effects of developmental treatments or ΔTL on days to pass discrimination learning; nor any significant correlations with CORT variables (see Supplementary Analysis).

25 of 31 birds completed the partial reinforcement phase in the minimum possible time (2 days), with the remainder taking 3, 4 or 5 days. As there was little variation in time to complete this phase, we did not analyse it further.

### Judgement bias testing: overall performance

All birds maintained discrimination during the testing phase (comparing the latencies of POS and NEG stimuli pooled from the 4 days of testing, Mann–Whitney tests, all *P* < 0.05) and therefore all 31 birds were retained for analyses. Descriptive statistics for performance in the test phase are presented in Table 1. The per-bird pessimism index had a mean of 0.47, standard deviation of 0.22, and range 0.08 (very ‘optimistic’) to 0.86 (very ‘pessimistic’).

**Table 1:** Descriptive statistics for stimuli in the judgement bias testing stage

	Stimulus				
	POS	NEARPOS	MID	NEARNEG	NEG
Trials per bird	24	8	8	8	24
Percentage probed within 60 s (mean ± per bird SD)	96.7 ± 5.5%	79.0 ± 20.7%	67.7 ± 29.0%	38.7 ± 27.5%	25.1 ± 22.1%
Latency to probe (s) (mean ± SD)	5.51 ± 11.39	15.91 ± 23.55	23.17 ± 26.84	39.79 ± 27.13	48.62 ± 22.27

### Judgement bias testing: familial resemblance

**Fig. 1:**
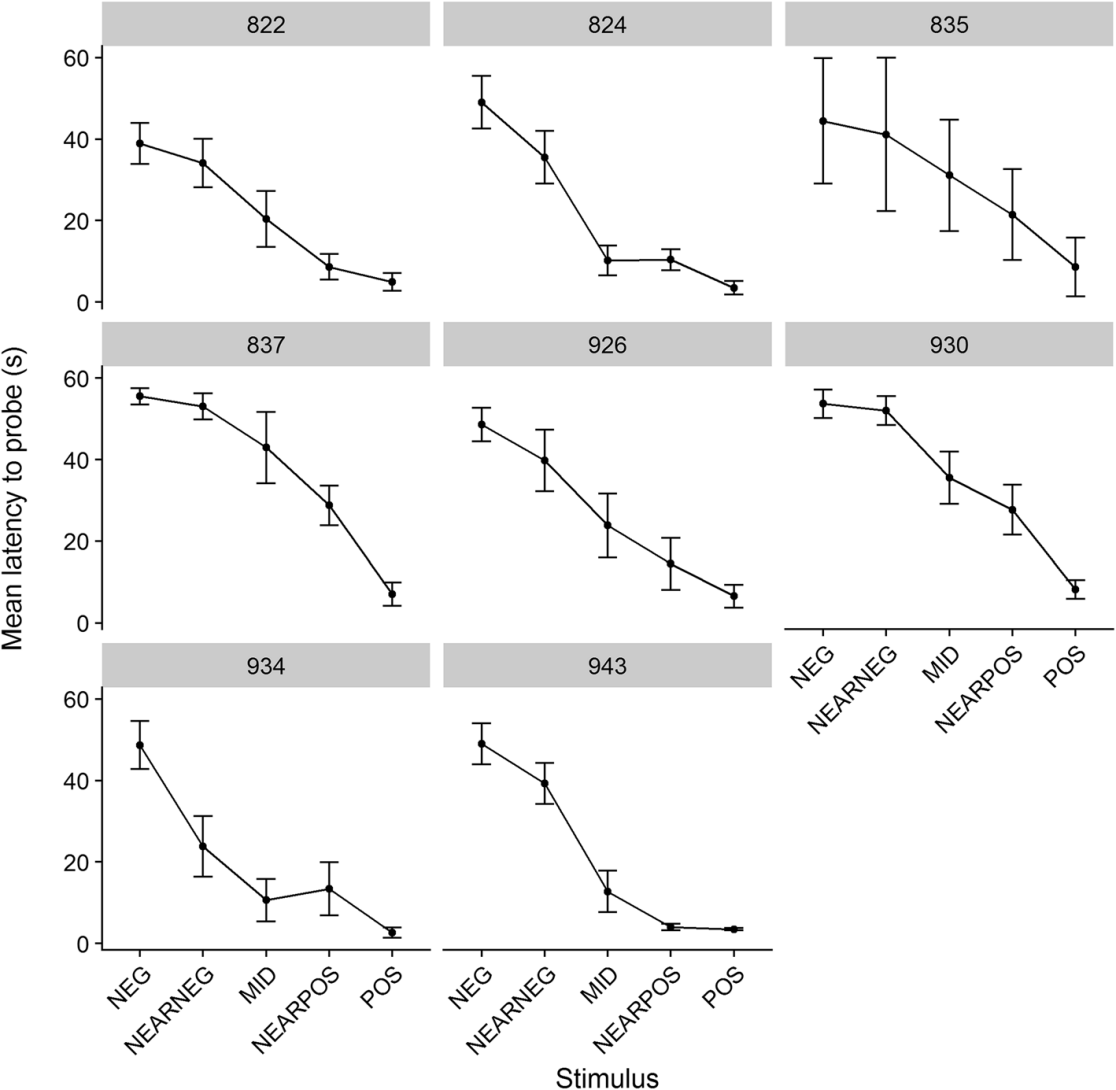
Mean latencies to probe by stimulus and natal family. Families are labelled by the number of the nest box from which they came. Error bars represent plus/minus one between-bird standard error

We first compared models containing a random effect of bird nested within natal family to models where family was omitted. For latency to probe the learned stimuli, the sole fixed predictor was valence (POS or NEG). The family level variance was estimated at zero, and the model fit was not improved by the inclusion of family (*χ*^2^ = 0.00, *P* = 1.00). For latency to probe the ambiguous stimuli, the fixed predictors were valence of stimulus (NEARPOS, MID, NEARNEG), and mean latency to probe the learned stimuli. The family level variance was estimated at 0.12, around 9% of the total variance. Inclusion of family significantly improved model fit for the latency to probe the ambiguous stimuli (*χ*^2^ = 6.46, *P* = 0.01). Visually, the shape of the latency gradient from the negative to positive stimulus via the three levels of intermediate was somewhat different for the different natal families (Fig. 1).

### Judgement bias testing: developmental treatments and telomere attrition

**Fig. 2:**
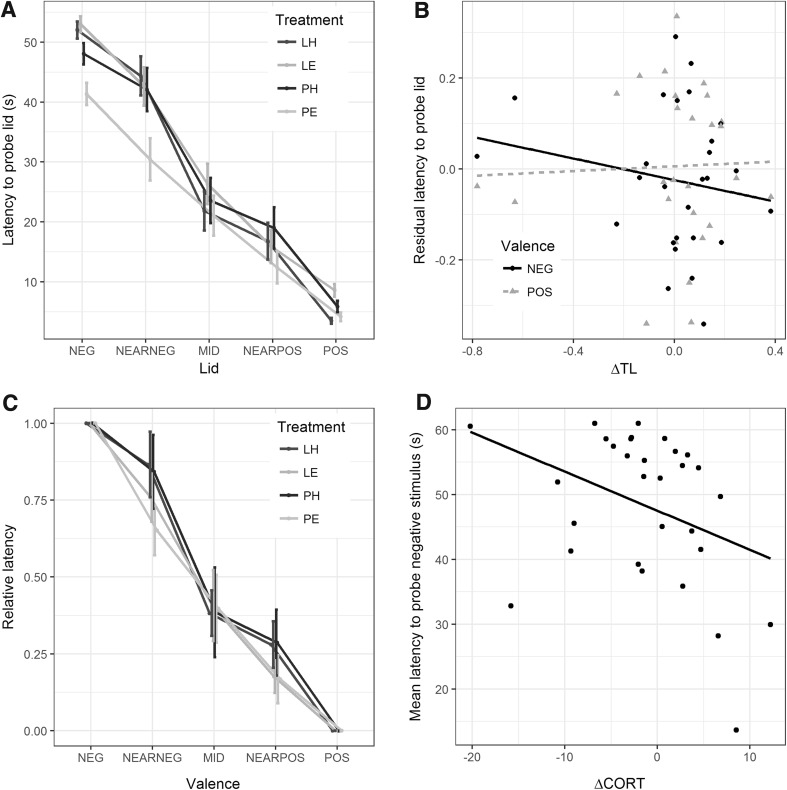
Summary of main results. **a** Mean latencies to probe all stimuli during the test phase, by developmental treatment group. Error bars represent between-bird standard errors. **b** Residual latency to probe learned stimuli, after controlling for developmental treatments, against developmental telomere change (ΔTL), by valence of stimulus. Note that each bird appears twice on this figure, represented by their mean residual latency to probe the positive stimulus, and their mean residual latency to probe the negative stimulus. **c** Mean latencies to probe stimuli relative to mean latency to probe the negative stimulus (represented as 0) and mean latency to probe the positive stimulus (represented as 1), by developmental treatment group. The formula for the relative latency to probe the ambiguous stimuli is RL = (latency − mean NEG)/(mean POS − mean NEG), where mean NEG is that bird’s mean latency to probe NEG and mean POS is that bird’s latency to probe POS. Error bars represent between-bird standard errors. **d** Mean latency to probe negative learned stimulus against ΔCORT. Each data point represents a bird, and the line represents a simple linear fit

Our first model used the learned stimuli (POS and NEG) trials, with amount, effort, ΔTL and relevant interactions as the fixed predictors (Table 2). As expected, there was a strong main effect of valence, with POS lids probed more quickly than NEG lids. In terms of developmental treatments, there was a significant three-way interaction between valence, amount and effort. This arose because the Plenty-Easy group showed shorter latencies than all other treatment groups towards the NEG stimulus (Fig. 2a). The Plenty-Easy group had the highest body condition (weight for skeletal size; means ± sd: Plenty-Easy: 7.07 ± 10.04, Lean-Easy: 3.87 ± 4.41; Plenty-Hard: 0.18 ± 3.73; Lean-Hard: − 0.10 ± 5.62), and body condition was negatively correlated with mean latency to probe the negative learned stimulus (*r* = − 0.43, *P* = 0.02). However, even after adding body condition to the model as an additional fixed predictor, the three-way interaction between valence, Amount and Effort remained significant (*t*_1269_ = 2.41, *P* = 0.02).

**Table 2:** Summary of linear mixed model predicting logged latencies to probe learned stimuli (NEG, POS) during the test phase, by experimental treatments and ΔTL

Variable	Parameter estimate	Standard error	*t*	*df*	*P* value
Valence	− 2.39	0.04	− 56.72	1269	< 0.001*
Amount	0.09	0.08	1.15	28.19	0.26
Effort	− 0.07	0.08	− 0.87	29.45	0.39
ΔTL	− 0.20	0.37	− 0.53	32.19	0.60
Amount × effort	0.04	0.07	0.65	25.53	0.52
Amount × valence	− 0.09	0.05	− 2.00	1269	0.046*
Effort × valence	0.20	0.05	4.16	1269	< 0.001*
ΔTL × valence	− 0.56	0.21	− 2.59	1269	0.01*
Amount × effort × valence	0.10	0.04	2.41	1269	0.02*

*P < 0.05

In addition to the interactive effect of the developmental treatments, there was a significant interaction between ΔTL and valence. After controlling for developmental treatments, for the NEG stimulus, latency to probe increased with increasing ΔTL, whereas for the POS stimulus, latency to probe decreased with increasing ΔTL (Fig. 2b). From Fig. 1b, it appears that this result may be driven by just two birds, the two with outlying ΔTL values of − 0.5 or less. To verify this, we calculated DFBETA, which is the standardised change in the parameter estimate of interest (here, the interaction between valence and ΔTL) when the values in question are left out (i.e. a DFBETA of 1 means the omission of that bird would change the parameter estimate by one standard error). In fact, neither of the two outlying birds was excessively influential for the parameter estimate in question: the omission of the first would actually make the effect stronger (DFBETA = − 0.60), whilst omission of the second (DFBETA = 0.06) would make little difference. Thus, these two birds are not the sole cause of the interaction between valence and ΔTL.

We next tested for effects of developmental treatments and ΔTL on latency to probe the ambiguous stimuli, after controlling for mean latency to probe the learned stimuli. In this model (Table 3), there were significant effects of mean learned latency (birds faster to probe the learned stimuli were also faster to probe the ambiguous stimuli), and of valence (the more similar to POS the lid, the shorter the average latency). There were no significant main effects or interactions involving the developmental treatments or ΔTL (Fig. 2c).

**Table 3:** Summary of linear mixed model predicting logged latencies to probe ambiguous stimuli (NEARNEG, MID, NEARPOS) during the test phase, by experimental treatments and ΔTL

Variable	Parameter estimate	Standard error	*t*	*df*	*P* value
Mean latency learned	0.09	0.01	6.71	19.70	< 0.001*
Valence	− 0.68	0.11	− 6.46	621	< 0.001*
Amount	− 0.04	0.37	− 0.12	186.79	0.91
Effort	− 0.12	0.38	− 0.32	205.97	0.75
ΔTL	− 0.06	0.68	− 0.09	177.93	0.93
Amount × effort	0.03	0.51	0.06	195.76	0.95
Amount × valence	0.12	0.15	0.80	621	0.42
Effort × valence	− 0.02	0.16	− 0.14	621	0.89
ΔTL × valence	− 0.11	0.27	− 0.40	621	0.69
Amount × effort × valence	− 0.02	0.21	− 0.09	621	0.93

*P < 0.05

### Judgement bias testing: relationships with CORT variables

The matrix of correlations between the CORT variables and performance on the judgement bias task is shown in Table 4. Only ΔCORT showed any evidence of association with judgement bias task performance, being marginally significantly associated with the mean negative learned latency (Fig. 2d), and marginally non-significantly associated with the pessimism index. Birds whose CORT levels returned more toward baseline between 15 and 30 min were faster to probe negative learned stimuli, and tended to be less pessimistic.

**Table 4:** Correlations between CORT variables and judgement bias performance

	Baseline CORT	Peak CORT	ΔCORT
Mean negative learned latency	0.08 (0.67)	− 0.11 (0.58)	− 0.38 (0.04*)
Mean positive learned latency	− 0.24 (0.20)	0.02 (0.93)	− 0.19 (0.32)
Pessimism index	− 0.14 (0.45)	0.26 (0.18)	− 0.36 (0.05)

*P* values are in parenthesis. Correlations involving Peak CORT are partial controlling for baseline CORT; those involving ΔCORT are partial controlling for CORT at 15 min

*P < 0.05

## Discussion

The primary aims of this study were (1) to conceptually replicate the prior findings of Bateson et al. (2015) concerning familial effects, and effects of early-life adversity and developmental telomere attrition on judgement bias in European starlings; and (2) to relate individual differences in the corticosterone response to acute stress to individual differences in judgement bias performance. In terms of objective (1), our findings were in most cases not the ones that the earlier study led us to predict. In the earlier study, early-life conditions affected latency to probe the negative learned stimulus, with the group that had experienced more adversity and slower weight gain during development probing the negative stimulus fastest. In the present study, we found an effect of early-life adversity on latency to probe the negative learned stimulus, but in our case, the birds with the most benign early conditions, and the fastest weight gain during development, were the ones that were fastest to probe. We also found an association between developmental telomere attrition and latency to probe the negative learned stimulus, with birds that had experienced greater telomere attrition faster to probe. This was reminiscent of Bateson et al. (2015) findings in that the developmental telomere attrition effect was in the opposite direction to that of the adverse developmental treatments, and birds with greater attrition probed faster, suggesting greater ‘desperation’ or boldness. However, the significant developmental telomere attrition result reported in the earlier paper was on latency to probe the ambiguous stimuli, whereas here it concerned the latency to probe the negative learned stimulus. The difference between our findings and those of Bateson et al. (2015), in relation to developmental history and developmental telomere attrition, is not that they found significant results whilst ours were null. Rather, both studies found significant results that are not easy to relate to one another. This illustrates the fact that early-life effects are complex, with their magnitude and even direction potentially depending on the exact nature of the early adversity, and the age at follow-up; and also that within judgement bias tasks, there can be independent influences on behaviour towards the learned stimuli, and on judgement bias itself.

The assumptions of the judgement bias paradigm were met in our experiment. The NEG stimulus produced a significantly greater latency to probe than the POS stimulus for every individual bird. Latencies to probe the ambiguous stimuli were intermediate between NEG and POS, with a gradient of latency from NEARPOS through MID to NEARNEG. Nonetheless, the key judgement bias result of the earlier study, concerning behaviour towards the ambiguous stimuli in particular, was not replicated here. We found that neither developmental treatment nor developmental telomere attrition significantly predicted behaviour towards the ambiguous stimuli once individual differences in speed to probe the learned stimuli were controlled for. The only result from the earlier paper that we closely replicated was that there were small natal-family effects on behaviour toward the ambiguous stimuli (9% in this study, 6% in Bateson et al. 2015); but no natal family effects at all on latency to probe the learned stimuli. Given that the present birds were taken from their natal nests on day 5 post-hatching, and all siblings rose in different treatments, these modest familial effects must represent genetic variation, maternal effects, or very early environmental influences.

In terms of objective (2), we found some evidence that individual differences in the response to acute stress may be associated with behaviour in the judgement bias task. Specifically, birds whose CORT levels returned more toward baseline between 15 and 30 min (i.e. with more negative values of ΔCORT) were slower to probe negative stimuli. A more negative value of ΔCORT has been interpreted as evidence of stronger negative feedback within the HPA axis, and a shorter duration of the stress response (Andrews et al. 2017). This means that birds with more negative values of ΔCORT would, if anything, be exposed to less total CORT in the course of a stress response. Thus, birds that tend to have a smaller total exposure to CORT appear to be more cautious to probe a stimulus that has been placed into their cage. The direction of this association is not intuitive. Moreover, it is not the association we predicted on the basis of previous experimental work (Enkel et al. 2010; Iyasere et al. 2017). Those experiments suggest that higher circulating CORT induces greater pessimism. On this basis, we predicted CORT relationships with behaviour towards the ambiguous stimuli after controlling for speed to probe the learned stimuli. We should note that the CORT measurements were taken 3 months prior to the judgement bias experiment, and previous research in birds has not always found individuals to be consistent over time, especially in baseline CORT (see Cockrem and Silverin 2002; Angelier et al. 2010; Ouyang et al. 2011; Rensel and Schoech 2011; Baugh et al. 2014; Lendvai et al. 2015). Thus, we view the observed correlations between behaviour and ΔCORT as exploratory observations in need of further confirmation.

Our judgement bias paradigm was different from that of Bateson et al. (2015) in that the negative stimulus here was the absence of reward, rather than a quinine mealworm. This means that our experiment is not a direct replication of the earlier one. However, we feel the change in protocol alone is unlikely to explain the difference in outcome. The birds in the earlier study continued to attack the negative lids, albeit with slower latencies than the positive. The pattern of latencies shown in Fig. 1a of the present paper (fastest to the positive learned stimulus, slowest to the negative learned stimulus, with the three ambiguous stimuli forming a continuum in between) was similar to that found in the earlier study. Moreover, there was variation between birds in how the intermediate stimuli were treated (as shown in Fig. 1). It was just not related to our main predictors.

The developmental manipulations in the present study were also different from that used in the earlier study (Bateson et al. 2015). There were good reasons for this: we designed the current manipulation to de-confound variation in food supply and in begging effort, and to minimise within-group variation in experience. However, a widespread conclusion in developmental programming research is that small changes to the nature, timing, or severity of developmental treatments can produce different or even opposite effects on the adult phenotype (Love and Williams 2008; Kriengwatana et al. 2014). In our own work, for example, we have observed different types of early-life adversity producing both lower and higher long-term adult body weights (Andrews et al. 2015; Dunn et al. 2018). Thus, it may be that our hand-rearing paradigm, rather than being a more controlled model of a brood-size manipulation in the wild, actually has qualitatively different effects. Moreover, the birds in this experiment came into human captivity earlier (day 5) than those of Bateson et al. (2015; day 15). Though hand-rearing by humans does not affect basic cognitive performance in the starling, there is some evidence it might produce changes in decision-making normally associated with reduced developmental stress (Feenders and Bateson 2013). To the extent that the present birds were hand-reared for more of their development than the earlier cohort, we might therefore expect a different baseline levels of emotionally mediated responding, with possible implications for the ability to detect significant judgement bias differences within the cohort. However, as mentioned above, the overall pattern of responding was similar to that in the study by Bateson et al. (2015), and there was substantial variation in ‘optimism’ and ‘pessimism’ within the present cohort.

We should also note that the birds were somewhat older in the present experiment (150–250 days at beginning of experiment) than the earlier study (94 days). This is potentially consequential. Some early-life effects resolve with time, being detectable in juveniles but not adults (Lendvai et al. 2009; Crino et al. 2014). Other effects may be delayed in appearance, leading to different early-life predictors being important at different ages. For example, in the current cohort of birds, amount of food received in early life was related to body weight close to the time of fledging, but this effect resolved, and later in adulthood, early-life begging effort became a strong predictor of adult body weight (Dunn et al. 2018). Effects can even change direction with age: in male rodents, maternal separation or deprivation appears to enhance hippocampal neurogenesis in the juvenile period, but suppress it in the adult period (Loi et al. 2014). Only repeated assessment of judgement bias at different ages in the same individuals would establish whether resolution with age explains the differences between our findings and those of Bateson et al. (2015). However, effects of nestling conditions on behaviour can certainly endure at least as long as the ages of the birds in the present experiment, as we have found several times before with similar designs (Bloxham et al. 2014; Neville et al. 2017; Dunn et al. 2018).

A feature of both this study and that of Bateson et al. (2015) is that the developmental treatments were related to latency to probe the learned stimuli. This suggests that early experience affects behavioural inhibition when foraging in the starling. A Go/No Go judgement bias task is effectively the super-position of two different tasks: the difference in response latencies between the positive and negative learned stimuli is a measure of behavioural inhibition, and the relative response to the ambiguous stimuli is a measure of judgement bias under ambiguity. It is important not to conflate effects of experimental treatments on the behavioural inhibition part with effects on the judgement bias part. This is why our analysis strategy (unlike Gygax 2014) involves modelling the learned latencies first, and then the ambiguous latencies controlling for the learned latencies as a separate step. In Bateson et al. (2015), it was the birds from the large broods that were faster to probe the learned negative stimulus, suggesting that early adversity causes poorer response inhibition and/or greater food motivation in adulthood. In our study, unexpectedly, it was birds from the Plenty-Easy group, which had the most benign developmental conditions that showed the least response inhibition. We have elsewhere suggested that the birds from the Hard treatments in this cohort are not cognitively impaired, but rather are programmed to defend a low level of body fat: this explains long-term differences in their masses and marked differences in their foraging motivation when food is cheap (Dunn et al. 2018). This may relate to the greater inhibition observed here in the presence of the negative learned stimuli, though it is not clear why the Lean-Easy group pattern with the Lean-Hard group, rather than the Plenty-Easy group, in the present experiment.

Given that it is behaviour towards the ambiguous stimuli in judgement bias tasks that is the marker of affective state, we did not find any evidence that our early-life developmental treatments produced chronic negative affect in adulthood in these birds using this paradigm, only that they affected response inhibition and/or food motivation. However, we have elsewhere found evidence consistent with effects of the developmental treatments on affect, in the same cohort of birds at an older age, using an alternative paradigm based on successive negative and successive positive contrast effects, which are also considered markers of negative affect (Neville et al. 2017). Thus, to add to the complexity due to different manipulations and different timings, it may be that some tasks are more suitable than others for recovering subtle emotional effects of early experience.

## Electronic supplementary material

Below is the link to the electronic supplementary material.

Supplementary material 1 (PDF 286 KB)
