# Supplementary material for: Developmental history and stress responsiveness are related to response inhibition, but not judgement bias, in a cohort of European starlings (Sturnus vulgaris)
Source: Anim Cogn. 2018 Nov 23;22(1):99–111. doi: 10.1007/s10071-018-1226-7 (PMC6327078; doi:10.1007/s10071-018-1226-7)
Supplement: Supplementary file 1 — Supplementary material 1 (PDF 286 KB) [file 10071_2018_1226_MOESM1_ESM.pdf]

Supplementary analyses for Gott et al. 'Developmental history and stress responsiveness are related to response inhibition, but not judgement bias, in a cohort of European starlings (*Sturnus vulgaris*)'

Effects of sex and body condition on latency to probe

To examine the effects of sex on latency to probe, and to establish whether it was important to control for sex in the main analyses, we fitted linear mixed models with latency to probe as the outcome (table S1). The first model contained sex, valence and their interaction as the fixed predictors, and included only the trials with the learned lids. The second model contained sex, valence, and the mean latency to probe the learned lids as fixed predictors, and included only trials with the ambiguous lids. Both models contained nested random effects of bird and natal family. There were no significant main effects or interactions involving sex.

Model	Variable	Parameter estimate	Standard error	t	df	P-value
1	Valence	-2.32	0.04	-57.44	1457	<0.001
	Sex	0.005	0.09	0.06	34.40	0.95
	Valence*Sex	0.03	0.04	0.86	1457	0.39
2	Mean latency learned	0.08	0.01	8.73	24.81	<0.001
	Valence	-0.19	0.05	-3.60	713	<0.001
	Sex	0.13	0.13	1.02	254.05	0.31
	Valence*Sex	-0.01	0.05	-0.19	713	0.85

Table S1. Output from models testing for effects of sex on latency to probe in the main experimental trials.

We also fitted mixed models with latency to probe as the outcome, and body condition as a fixed predictor, in interaction with valence (table S2). The first model was restricted to trials involving the learned stimuli, and the second to trials involving the ambiguous stimuli, controlling for mean speed to probe the learned stimuli. Both models contained nested random effects of bird and natal family. In the first model, there was a significant association between latency to probe and body condition, with heavier birds probing faster.

Model	Variable	Parameter estimate	Standard error	t	df	P-value
1	Valence	-2.34	0.04	-53.34	1457	<0.001
	Body condition	-0.03	0.01	-2.63	34.13	0.01
	Valence*BC	0.005	0.006	0.84	1457	0.40
2	Mean latency learned	0.08	0.01	7.16	26.89	<0.001
	Valence	-0.21	0.06	-3.54	713	<0.001
	Body condition	-0.02	0.02	-1.03	177.59	0.30
	Valence*BC	0.004	0.008	0.56	713	0.57

Table S2. Output from models testing for effects of body condition on latency to probe in the main experimental trials.

Lid-probing training

We fitted a mixed model with number of trials to successfully probe lids as the outcome (table S3). The fixed predictors were the developmental treatments plus ΔTL; and there was a random effect of natal family. None of the variables were significant predictors of trials to pass lid-probing training. We also correlated the CORT variables with number of trials to successfully probe lids; no correlation was significant (baseline CORT: r = -0.09, p = 0.63; peak CORT: r = 0.29, p = 0.13; ΔCORT: r = 0.14, p =0.48). There were also no significant associations between sex or body condition and trials to pass lid-probing (not shown).

Variable	Parameter estimate	Standard error	t	df	P-value
Amount	0.01	0.06	0.20	27	0.85
Effort	0.04	0.07	0.50	27	0.62
Amount*Effort	0.11	0.06	1.65	27	0.11
ΔTL	-0.24	0.33	-0.72	27	0.48

Table S3. Output from model predicting trials to pass lid-probing training from developmental treatments plus ΔTL.

Discrimination training

We fitted a mixed model with number of days to pass discrimination training as the outcome, and as predictors the developmental treatments plus ΔTL, and a random effect of natal family (table S4). There were no significant predictors in this model. We also correlated the CORT variables with number of trials to successfully probe lids; no correlation was significant (baseline CORT: r = -0.11, p = 0.55; peak CORT: r = -0.16, p = 0.41; ΔCORT: r = 0.27, p =0.15). There were no significant associations between sex or body condition and days to pass discrimination (not shown).

Variable	Parameter estimate	Standard error	T	df	P-value
Amount	0.02	0.31	0.07	27	0.95
Effort	0.22	0.31	0.70	27	0.49
Amount*Effort	0.18	0.28	0.63	27	0.54
ΔTL	-1.13	1.43	-0.79	27	0.44

Table S4. Output from model predicting days to pass discrimination training from developmental treatments plus ΔTL.
